# The immune response to 6-monthly versus annual standard dose inactivated trivalent influenza vaccination in older people: study protocol for a randomised clinical trial

**DOI:** 10.1186/s13063-017-1808-8

**Published:** 2017-02-10

**Authors:** Barnaby Young, Sapna Sadarangani, Haur Sen Yew, Chee Fu Yung, Yee Sin Leo, Mark I-Cheng Chen, Annelies Wilder-Smith

**Affiliations:** 1 Institute of Infectious Diseases and Epidemiology (IIDE), Tan Tock Seng Hospital, 11 Jalan, Singapore, 308433 Singapore; 2 Lee Kong Chian School of Medicine, Nanyang Technological University, 11 Mandalay Road, Singapore, 308232 Singapore; 3 Infectious Diseases Service, KK Women’s and Children’s Hospital, 100 Bukit Timah Road, Singapore, 229899 Singapore; 4 Saw Swee Hock School of Public Health, Thair Foundation Building, National University of Singapore, 12 Science Drive 2, Singapore, 117549 Singapore

**Keywords:** Influenza, Vaccine, Antibody persistence, Tropics, Haemagglutination inhibition, Older people

## Abstract

**Background:**

The seasonal influenza vaccine is less effective in older people and a single dose is unlikely to provide the year-round protection necessary for tropical climates which have year-round influenza virus activity. This study aims to assess the effect of a trivalent inactivated influenza vaccine (IIV3) booster at 180 days on haemagglutination-inhibition (HI) antibody titres for each of the influenza strains present in the administered vaccine in older people aged 65 years or above in Singapore.

**Methods/design:**

This is a single-centre, randomised, observer-blind, active-comparator controlled, parallel-group, phase IV trial in 200 adults aged 65 years or older. Study participants will be assigned to one of two groups in a 1:1 ratio and followed for 1 year, with five scheduled visits. The control group will receive IIV3 at day 1, and an active comparator (Tetanus-diphtheria-pertussis vaccine) at day 180. Participants in the experimental group will receive IIV3 containing the same strains at day 1 and day 180. Endpoints are immunological, and include measures of HI titres, microneutralisation titres (MN) and cell-mediated immunity from first vaccination up to day 360.

**Discussion:**

If superiority of 6-monthly influenza vaccination is demonstrated, this study could form the basis for a larger clinical trial with influenza infection as the primary endpoint.

**Trial registration:**

ClinicalTrials.gov, ID: NCT02655874. Registered on 12 January 2016.

**Electronic supplementary material:**

The online version of this article (doi:10.1186/s13063-017-1808-8) contains supplementary material, which is available to authorized users.

## Background

Influenza is popularly characterised as an infection acquired during the cold, winter months in temperate climates. Tropical and subtropical countries also have a significant burden of disease from influenza but the epidemiology is much more variable [1, 2]. This includes multiple annual influenza seasons and year-round virus activity [3, 4]. The implications of this for influenza vaccination schedules have not been tested in randomised clinical trials but are likely to be important in older people and in other populations with impaired vaccine responses.

Year-round influenza virus activity implies that an effective vaccine must also provide year-round protection. However, the duration of protection provided by the influenza vaccine has not been well established. No clinical studies of vaccine efficacy or effectiveness are available from countries with year-round influenza activity, but over the course of a single winter season declining vaccine effectiveness has been reported from test-negative case control studies in Europe and Australia [5–7]. For example, in Spain vaccine effectiveness was 61% (95% CI 5 to 84) in the first 100 days after vaccination, 42% (95% CI −39 to 75) between 100 and 119 days, and 0% thereafter. This decline in effectiveness mainly affected older people aged over 65 years [6].

In response to these reports, the I-MOVE multicentre, test-negative, case-control study reviewed data collected from more than 11,000 seasonal influenza infections over 2010–2015 in eight European countries [8]. Vaccine effectiveness against influenza A/H3N2 and B infection declined across all age groups as the winter season progressed. This decline was most marked in older people, with little vaccine effectiveness evident by day 140 (subtype A/H3N2) or day 200 (influenza B). Surprisingly, vaccine effectiveness appeared to be maintained (over 200 days) for subtype A/H1N1. The authors hypothesise that this may reflect the homogeneity of the A/H1N1 strain (pdm2009) across the period of the study, with repeated vaccination (or infections) boosting strain-specific antibody titres.

As an alternative to clinical effectiveness, the trajectory of immune responses after vaccination can be used to estimate the duration of protection. The haemagglutination inhibition (HI) titre is the major immune correlate of protection, and is used by regulatory agencies, such as the Food and Drug Administration (FDA) and the European Medicines Agency (EMA), for influenza vaccine licensing. A titre ≥1:40 is estimated to offer 50% protection against infection, and this threshold is conventionally labelled as ‘seroprotection’ [9]. A systematic review of vaccine antibody persistence studies, performed by the study team in preparation for this trial, suggested that in older people there is a steady decline in HI titres, and at between 180 and 360 days titres return to the pre-vaccination level [10]. Studies have identified increasing age and lower pre-existing HI titres as predicting reduced duration of antibody persistence, while the impact of comorbidities, such as diabetes, on persistence is less clear [11, 12].

### Primary objective

The study aims to assess the effect of a trivalent inactivated influenza vaccine (IIV3) booster at 180 days on HI antibody titres for each of the influenza strains present in the administered vaccine in adults, aged 65 years and older.

### Secondary objectives

- To compare the antibody response (HI and microneutralisation (MN) titres) at 360 days after IIV3 and IIV3 + booster
- To compare the immune response between primary and booster IIV3 vaccination
- To assess the safety of an IIV3 booster at 180 days

We hypothesise a booster injection of seasonal IIV3 at 180 days after primary vaccination improves year-round seroprotection against influenza infection in older people aged 65 years and above.

## Methods/design

### Summary of study design

**Fig. 1:**
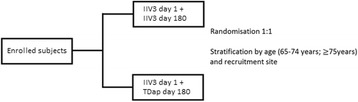
Stratified randomisation by age at enrolment day 1

**Fig. 2:**
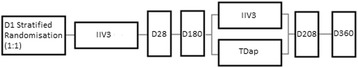
Study timeline from day 1 (D1) to day 360 (D360)

This is a single-centre, randomised, observer-blind, active-comparator controlled, parallel-group, phase IV trial in 200 adults aged 65 years or older. Each study participant who meets eligibility criteria and signs the Informed Consent Form will be allocated to one of two groups in a 1:1 ratio (Fig. 1). Participants will be followed for 1 year, with five scheduled visits. All participants will provide blood samples for immunogenicity assessment at days 1 (pre-injection), 28, 180 (pre-injection), 208, and 360 (Fig. 2).

Solicited and unsolicited adverse events (AEs) will be collected for all participants for 7 days after each vaccination. All serious adverse events (SAEs) will be collected for 28 days after each vaccination. SAEs that are considered related to vaccination and/or study procedures will be monitored until resolution or stable. Participants will also be contacted 2-weekly as surveillance for clinical endpoints, including influenza-like illnesses (ILI) and polymerase chain reaction (PCR)-confirmed influenza.

### Randomisation

A web-based, computer-generated randomisation code will assign participants using a random permuted block method. Allocation will be stratified by age (65 years or under to 74 years, 75 years or older). Age is a significant predictor of response to vaccination, and the proportion of participants recruited who are older than 75 years may be small.

### Blinding

To minimise the potential for unintentional bias an observer-blind procedure will be followed. The participant, investigators, study staff members, and laboratory personnel will all be blinded to group assignment. One or more unblinded vaccine administrators will perform randomisation and will be responsible for both preparing and administering the blinded vaccine. This person will not be authorised to collect any safety data or to perform any other study procedures. This person will not provide any information about the contents of the syringe to study participants or personnel, and will not allow anyone to see the content of the syringe. Blinded study personnel will not be present during product preparation and administration. The vaccine will be administered in a private setting with no observers present. Contents of the syringe will be masked with an opaque tape to ensure participant blinding to group allocation. The unblinded staff will ensure that vaccination documents revealing participant group assignment are stored in a secure place to which only they have access.

### Unblinding procedures

The vaccine blind will be broken by the investigator in the event of an SAE if identification of the vaccine received could influence treatment of the SAE, or in the event of death or a life-threatening SAE where the investigator’s causality assessment is ‘related’ to the trial product. Vaccine blind will also be broken at the request of the health authority in the event of an SAE. Code-breaking will be limited, when possible, to the participant(s) experiencing the SAE. Any intentional or unintentional code breaking will be reported to the Domain Specific Review Boards (DSRB).

### Potential risks

As with any vaccination, participants may experience pain, swelling, redness or itching at the injection site. Rarely, a participant may experience an allergic reaction which produces rash, urticaria or difficulty breathing. Serious allergic reactions that can be life-threatening may occur. The incidence of severe adverse reactions are low with the trial vaccines. An increased risk of SAEs has not been reported from studies of repeated vaccination at 1 year or earlier. The risk of nonsevere local and systemic adverse reactions may be increased with repeated vaccination as a result of increased reactogenicity from pre-existing immunity. Blood taking is a common medical procedure with very low risk of complications, such as prolonged bleeding or infection, when standard procedures are followed. Risks of breaches in trial participant confidentiality will be ameliorated by following the Singapore Guideline for Good Clinical Practice (SGGCP) and the DSRB-approved study protocol.

### Potential benefits

There is a possibility that the booster vaccine administered in the experimental arm will prevent an influenza infection that might otherwise have occurred. There is also a possibility that the vaccine administered in the control arm will prevent a tetanus, diphtheria or pertussis episode that might otherwise have occurred. Participants will not be paid for participation in this study, but will be reimbursed for their time and travel costs.

### Study population

Two hundred participants will be enrolled (approximately 100 per group). The study population will be drawn from people attending outpatient services at Tan Tock Seng Hospital, Singapore and healthy volunteers from senior activity centres and community clubs in Singapore. There are no participant restrictions based on sex or race. A complete review of the inclusion and exclusion criteria for eligibility will be performed. This will entail a face-to-face interview with the potential participant and screening of electronic medical records. The potential participant will be provided with information about the trial and written informed consent obtained. No procedure or treatment associated with the study will be performed before obtaining written informed consent.

### Inclusion criteria

Participants must meet all of the inclusion criteria to participate in this study:

- Age 65 years or older on the day of inclusion
- No influenza vaccination in the previous 10 months
- No tetanus, diphtheria or pertussis vaccine in the previous 1 year
- No virologically confirmed influenza infection in the previous 10 months
- Participant able to provide written informed consent
- Able to attend all scheduled visits and comply with all trial procedures

### Exclusion criteria

Participants meeting any of the exclusion criteria at baseline will be excluded from participation:

- Participation in the 4 weeks preceding the first trial vaccination or participation during the present trial period in another trial investigating a vaccine, drug, medical device, or medical procedure
- History of a life-threatening reaction to the vaccine used in the trial, or to a vaccine containing any of the same substances
- Known systemic hypersensitivity to any of the vaccine components, including:
  - Egg protein (eggs or egg products)
  - Chicken products
  - Formaldehyde
  - Neomycin or kanamycin
  - Octoxinol 9 (Triton X-100)
  - Cetyltrimethylammonium bromide (CTAB)
  - Thiomersal
- History of Guillain-Barré syndrome (GBS) within 6 weeks following previous influenza vaccination
- Acute respiratory infection (ARI) on the day of enrolment
- Moderate or severe acute illness/infection (according to investigator judgement) on the day of vaccination, or febrile illness (temperature ≥37.5 °C). A prospective participant should not be included in the study until the condition has resolved or the febrile event has subsided
- Self-reported thrombocytopenia, contraindicating intramuscular (IM) vaccination
- Known or suspected congenital or acquired immunodeficiency; or receipt of immunosuppressive therapy, such as anticancer chemotherapy or radiation therapy, within the preceding 6 months; or long-term systemic corticosteroid therapy (prednisolone ≥7.5 mg/day or equivalent for more than two consecutive weeks within the past 3 months)
- Chronic illness that, in the opinion of the investigator, is at a stage where it might interfere with trial conduct or completion
- Deprivation of freedom by an administrative or court order, or in an emergency setting, or hospitalised involuntarily
- Current alcohol abuse or drug addiction that might interfere with the ability to comply with trial procedures in the opinion of the investigator
- Women of childbearing age will be excluded from the trial

### Participant withdrawal

Participants will be informed that they have the right to withdraw from the trial at any time. Participants who are withdrawn will not be replaced. If withdrawal occurs within 28 days after vaccination, and if agreeable, the participant will continue to be contacted by telephone at expected visits to confirm AE or SAE status.

The investigator will discontinue vaccination if the participant experiences any SAE related to the administration of the previous vaccine, including an anaphylactic or other significant allergic reaction. In addition, a participant may be withdrawn from the study if, in the investigator’s judgement, there are safety concerns, or significant noncompliance with the study protocol. If a participant experiences an AE or SAE considered by the investigator to be related to vaccination, the participant will be followed until the condition resolves or becomes stable.

### Primary outcome

The primary outcome is the percentage of participants with seroprotection (HI titre ≥1:40) at day 208 post primary vaccination for each of the influenza strains present in the administered influenza vaccine.

### Secondary outcomes

The secondary outcomes are as follows:

- Comparison by vaccination group of Geometric Mean Titres (GMTs) against homologous and heterologous influenza strains from day 208 to day 360
- Comparison by vaccination group of the Geometric Mean Ratio (GMR) of HI titres post versus pre-vaccination (day 1), against homologous and heterologous strains from day 208 to day 360
- Comparison by vaccination group of seroprotection rates (HI titre ≥1:40) against homologous and heterologous strains from day 208 to day 360
- Comparison by vaccination group of participants with seroconversion against homologous and heterologous strains from day 208 to day 360 (seroconversion is defined as a pre-vaccination (day 1) HI titre <10 and post-vaccination HI titre ≥40 or at least a four-fold increase in HI titres from a pre-vaccination HI titre ≥10)
- Comparison by vaccination group of MN titres against homologous and heterologous strains from day 208 to day 360
- Number of participants reporting ILI across vaccine groups from day 208 to day 360
- Number of participants with PCR-confirmed influenza across vaccine groups from day 208 to day 360
- Number of participants reporting healthcare utilisation (emergency room visits, unscheduled physician visits, and hospitalisations in each group) across vaccine groups from day 180 to day 360
- All-cause mortality rate, across vaccine groups from day 180 to day 360
- Frequency and severity of solicited local (injection site) and systemic AEs for 7 days post vaccination. The frequency of the following AEs will be reported per regulatory guidance (CPMP/BWP214/96): indurations larger than 50 mm in diameter and persisting for more than 3 days, ecchymosis, temperature >38 °C for 24 h or more, malaise, shivering
- Percentage of participants with SAE from day 1 to day 360

### Study schedule, visits and procedures

**Fig. 3:**
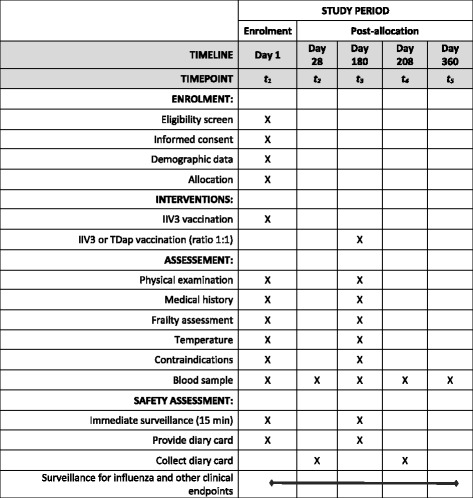
Standard Protocol Items: Recommendations for Interventional Trials (SPIRIT) flow diagram of study enrolment, interventions and assessments

The study schedule is presented in Fig. 3.

### Blood sample analysis

HI and MN antibody testing will be performed by collaborators at a central laboratory. Antibody assays will be performed using standard methods recommended by the World Health Organisation (WHO) [13]. As both assays are subject to considerable interlaboratory variability, assays will be conducted at a single, experienced laboratory using the same technique.

### Influenza surveillance

For all participants, there will be 2-weekly contact (telephone, home visit, or clinic visit) from enrolment to day 360. The method of contact can vary (e.g. telephone, text message, or email); however, there should be participant contact via telephone or face-to-face visit once a month. At each contact, participants will be asked regarding any symptoms meeting protocol definition of an acute respiratory illness, any unscheduled physician visits (e.g. general practitioner, polyclinic, emergency department) or hospitalisations since the previous contact. Participants will also be instructed to contact the study team if they develop any acute respiratory symptoms.

Study investigators will determine whether symptoms meet the European Centre for Disease Prevention and Control (ECDC) definition of an ARI:

1. Sudden onset of symptoms
2. And at least one of the following four systemic symptoms:
  Fever or feverishness
  Malaise
  Headache
  Myalgia
3. And at least one of the following respiratory symptoms:
  Cough
  Sore throat
  Shortness of breath
  Coryza

If symptoms meet the criteria for an ARI, a study team member will visit the participant to collect a nasopharyngeal swab within 5 days after onset of the illness. Influenza and other respiratory viral pathogens in nasopharyngeal swabs will be detected using a PCR assay. No additional blood samples will be collected during ARI episodes.

### Trial materials

All trial products will be dosed according to Singapore licenses. The vaccine formulation containing the most recent WHO recommendations for strain identity at the start of the trial will be used. Southern Hemisphere winter 2016 and Northern Hemisphere winter 2016/7 recommendations are as below, but may change during the trial:

- A/California/7/2009 (H1N1)pdm09-like virus
- A/Hong Kong/4801/2014 (H3N2)-like virus
- B/Brisbane/60/2008-like virus

Participants will receive one injection of 0.5 ml standard dose, inactivated IIV3 at days 1, and no or one injection at day 180 depending on group allocation. Participants will receive no or one injection of 0.5 ml Tetanus-diphtheria-pertussis vaccine (TDap) at 180 days depending on group allocation.

### Treatment compliance and specific restrictions

The following measures will ensure that the vaccine doses administered comply with those planned, and that any noncompliance is documented so that it can be accounted for in the data analyses. All vaccines will be administered by qualified trial personnel, the person in charge of product management will maintain accountability records of product delivery to the trial site, product inventory at the site, doses given to each participant, and the disposal of unused or wasted doses. Participants will be asked not to take any influenza vaccinations except as provided by the study team for the duration of the study. This restriction will not apply in the event of a national immunisation programme by the Ministry of Health for a pandemic influenza vaccine. There are no restrictions on other vaccines. If prescribed at the same visit they will be administered to a separate body site. There are no limitations on medications, herbs, vitamins, and mineral supplements while participating in the study.

### Safety measurements

AEs, SAEs and ‘Unanticipated Problems Involving Risk to Participants or Others’ (UPIRTSO) events are defined below. Events will be reviewed and graded by the principal investigator (PI) or other investigator. Only members of the study team blinded to participant group allocation will be involved in safety assessment. Severity will be classified using the Common Terminology for Adverse Events version 4.03. The relationship of the event to the study drug, and whether the event is an expected event or not, will be assessed using the list of AEs contained in the product summary of characteristics.

### Adverse event (AE)

An AE is any untoward medical occurrence in a participant administered a trial product. It does not necessarily have a causal relationship with this product. Pre-existing medical conditions are not reported as an AE; an AE may be a new illness, the worsening of a concomitant illness or an effect of vaccination. All AEs include serious and nonserious AEs. Surgical procedures are not AEs; they are the action taken to treat a medical condition. The condition leading to the surgical procedure is the AE, if it occurs during the trial period. AEs will be further categorised as solicited and unsolicited AEs.

A solicited AE is a reaction that is prelisted in the electronic Case Report Form (eCRF). It is defined by nature and time of onset post vaccination, and is considered to be related to vaccination. Unsolicited AEs are all observed AEs which do not fulfil the conditions for a solicited AE, as described above.

### Serious adverse events (SAEs)

A SAE is defined as any untoward medical occurrence that: results in death, is life-threatening (immediate risk of death), requires inpatient hospitalisation or prolongation of existing hospitalisation, results in persistent or significant disability/incapacity, results in congenital anomaly/birth defect, or is a medically important event. Medical and scientific judgment will be exercised in determining whether an event is an important medical event. An important medical event may not be immediately life-threatening and/or result in death or hospitalisation. However, if it is determined that the event may jeopardise the participant and/or may require intervention to prevent one of the other AE outcomes, the important medical event should be reported as serious.

### Collecting, recording and reporting of SAEs to the Health Sciences Authority (HSA)

All SAEs that are unexpected and related to the study drug will be reported to the HSA within 15 calendar days after initial notification to the PI. For fatal or life-threatening cases, the HSA will be notified as soon as possible, but no later than seven calendar days after first knowledge that a case qualifies. A complete report will then follow within eight additional calendar days.

### Collecting, recording and reporting of UPIRTSO events to the National Healthcare Group Domain Specific Review Boards (NHG DSRB)

Any events that are unexpected (in terms of severity or frequency), that can be reasonably attributed to the study drug and that may expose other participants to harm will be reported. A UPIRTSO event refers to problems, in general, that include any incident, experience, or outcome (including AEs) which meet all of the following criteria:

1. Unexpected, in terms of nature, severity, or frequency of the problem as described in the study documentation (e.g. protocol, consent documents, etc.)
2. Related or possibly related to participation in the research. Possibly related means that there is a reasonable possibility that the problem may have been caused by the procedures involved in the research, and
3. Risk of harm. This suggests that the research places participants or others at a greater risk of harm (including physical, psychological, economic, or social harm) than was previously known or recognised

For urgent reporting, all problems involving local deaths, whether related or not, should be reported immediately – within 24 h after first knowledge by the investigator. For expedited reporting, all other problems must be reported as soon as possible but not later than seven calendar days after first becoming known to the investigator.

### Safety assessment methods

To ensure participants’ safety, there will be an immediate post-vaccination surveillance period. Participants will be kept under observation for 15 min after each vaccination. AEs will not be actively solicited during this surveillance period. To collect solicited AEs, all participants will be provided with a safety diary card, a digital thermometer, and a flexible ruler and will be instructed how to use them. The following items will be recorded in the diary card on the day of vaccination and for the next 6 days or until resolution: daily temperature with the route by which it was taken; daily measurement or intensity grade of all other solicited injection site and systemic reactions; action taken for each event (e.g. medication), if any. To collect unsolicited AEs, all participants will be instructed to record any unsolicited AEs that may occur for 7 days after each vaccination. Space will be provided in the diary card for this purpose. AEs which are likely to be related to trial products, whether serious or not, will be followed up by the investigator until their complete disappearance or stabilisation. Information on all SAEs and UPIRTSO events will be collected and assessed throughout the trial, from enrolment until the final study visit (day 360).

### Safety monitoring plan

All vaccines used in this study are part of routine vaccination schedules. Safety data has been established from millions of administered doses. According to the WHO, the incidence of vaccine-related SAEs, such as anaphylaxis and GBS, is approximately 1 per million doses. The rate of mild, nonsevere AEs in clinical trials is similar between influenza vaccine and placebo. Vaccine-related SAEs are not expected in a clinical trial of this size. AEs will be monitored for the first 7 days after each vaccination. SAEs will be monitored for the first 28 days after each vaccination. An interim safety analysis will not be performed. The study team will review any UPIRTSO events and stop the trial if significant risk to participants from continuing in the trial is suspected.

### Data entry and storage

Paper Case Report Forms (CRFs) will be used for initial data collection. These will be transcribed to an eCRF developed with an independent web-based hosting facility called Research Electronic Data Capture (REDCap). The trial database will include information on demographics (age, gender), underlying illnesses, concomitant medications and vaccination history. AE and SAE data will also be entered. Paper documents will be maintained and stored in a locked office, with access restricted to study personnel. All electronic documents with participant information will be password-protected.

### Data quality assurance

A random sample of source documents and CRFs will be audited to substantiate the integrity of trial data entered into the electronic database.

### Sample size calculation

The following assumptions were made for determination of sample size expected to meet primary outcome:

1. Rate of seroprotection (HI antibody titre ≥1:40) on day 208 after vaccination is 60%
2. Proportion seroprotected at day 208 in the experimental group will be 80%
3. A loss to follow-up rate of 10%
4. A power (1 − *β*) of 80% to detect differences between the two primary study groups
5. A two-sided significance level (*α*) of 5%

The primary statistical null hypothesis is of no increase in seroprotection rates (HI antibody titre ≥1:40) at day 208 following booster IIV3 vaccination, compared to a control injection. Formally, the trial will test:

- H_0_: Seroprotection rate (booster) = seroprotection rate (control), versus
- H_A_: Seroprotection rate (booster) ≠ seroprotection rate (control)

A sample size of 164 (82 per group) will provide 80% power to test this hypothesis, based on approximations to the normal distribution. A total sample size of 200 (100 per group) has been chosen to allow for up to 15% dropout. This sample size will provide adequate power for a range of seroprotection rates assuming an increase in absolute seroprotection of at least 20%.

### Statistical analysis

The full analysis set (FAS) consists of all participants who received at least one injection. Participants will be analysed according to the group to which they were randomised. The per-protocol analysis set (PPS) is a subset of the FAS who have no major protocol violations. Participants presenting with at least one of the following protocol deviations will be excluded from the per protocol analysis: participant did not meet all protocol-specified inclusion criteria or met at least one of the protocol-specified exclusion criteria; participant did not complete the vaccination schedule; participant received a different vaccine than the one randomised to receive; participant did not receive vaccine within the protocol-determined time window; participant received additional seasonal trivalent influenza vaccines during the trial period; preparation and/or administration of the vaccine was not performed as per protocol.

The safety analysis set is defined as those participants who have received at least one vaccine. All participants will have their safety analysed according to the vaccine that they actually received. The FAS will be the primary analysis to infer superiority of a 180-day IIV3 booster vaccination compared to the active-comparator control in terms of the proportion of participants with day-208 HI antibody titres ≥1:40. This will be supported by per-protocol analysis. Analysis of secondary endpoints will use both the FAS and PPS.

Immunological data will be analysed per CHMP guidance (EMA/CHMP/VWP/457259/2014). Results will be presented by vaccine strain and will include GMTs (with 95% confidence intervals) and pre-/post-vaccination ratios (GMRs). Reverse cumulative distribution curves will also be provided, supplemented by tables presenting percentages of vaccinees with titres above a range of cut-off levels on a logarithmic scale.

### Confidentiality and retention of trial documents

All study findings and documents will be regarded as confidential. The investigators and other study personnel will not disclose such information without prior written approval from the PI. Participant confidentiality will be strictly maintained to the extent possible under the law and as required by the SGGCP. Identifiable information will be removed from any published data. Any electronic data records stored locally will be kept on a networked, password-protected, single computer within the Department of Infectious Diseases. The PI will keep any paper-based records, DSRB files or source documentation in a locked cabinet within the department. These records, electronic and physical, will be kept for a minimum of 6 years after trial completion, before being destroyed or erased as per SGGCP requirements.

### SPIRIT Checklist

Please see Additional file 1 for the SPIRIT Checklist

## Discussion

This clinical trial addresses a neglected issue in tropical countries. Demonstrating superior protection against influenza infection with a simple booster vaccination has the potential to offer a significant public health benefit. For example, in South East Asia alone, demographic estimates by the United Nations are for the number of persons aged 65 years and older to almost triple over the next 30 years, from 37.6 million in 2015, to 111.3 million in 2045 [14].

A 6-monthly booster schedule with standard dose inactivated trivalent vaccine is attractive due to its simplicity, low cost, and safety profile. This would support its implementation into clinical practice if proven to offer superior efficacy against influenza infection. Six-monthly vaccination coincides with the approximate periodicity of epidemics in countries with biannual seasons synchronous with the Northern and Southern Hemisphere winters. A 6-monthly schedule also coincides with WHO strain recommendations which, since 1999, have been made biannually [15]. Between hemisphere winters, a change in at least one of the strains in the trivalent vaccine has been recommended approximately half of the time (19/37 from 1999 till the SH 2017 recommendations). Repeat vaccinations are not associated with an increased risk of AEs, though data is not available for 6-monthly vaccination [16].

The limitations in older people of standard dose inactivated influenza vaccine are well understood [17]. Even if this study finds a significant immunological benefit from repeat vaccination at 6 months, this may not be the optimal solution for providing year-round protection. Alternative influenza vaccine formulations have been licensed for older people; for example, a high-dose vaccine with 60 μg of haemagglutinin (HA) per strain, and vaccines which include an immune adjuvant. These vaccines do offer superior short-term immunogenicity in older people, which in some studies persists for at least 6 months [18–20]. However, by 1 year, either no significant residual benefit is reported (adjuvanted vaccine), or data is not currently available (high-dose vaccine) [21, 22].

If evidence of significantly improved seroprotection by an IIV3 booster can be inferred from this study, we believe that it will provide the basis for a large-scale clinical trial in older people to determine whether this translates into reduced influenza infection rates.

### Trial status

This trial began recruiting participants in May 2016. Recruitment is expected to conclude by the end of 2016, with the final participant visit by the end of 2017.

## Additional file

Additional file 1: SPIRIT Checklist. (DOCX 37 kb)

## Abbreviations

AE: Adverse event
ARI: Acute respiratory infection
CRF: Case Report Form
DSRB: Domain Specific Review Boards
eCRF: Electronic Case Report Form
EMA: European Medicines Agency
FAS: Full analysis set
GBS: Guillain-Barré syndrome
HI: Haemagglutination inhibition
HSA: Health Sciences Authority
IIV3: Trivalent inactivated influenza vaccine
ILI: Influenza-like illnesses
IM: Intramuscular
MN: Microneutralisation
MOH: Ministry of Health
NHG: National Healthcare Group
PCR: Polymerase chain reaction
PPS: Per-protocol analysis set
PI: Principal investigator
SAE: Serious adverse event
SGGCP: Singapore Guideline for Good Clinical Practice
UPIRTSO: Unanticipated Problems Involving Risk to Participants or Others
VE: Vaccine effectiveness
WHO: World Health Organisation
